# Ischemic preconditioning does not alter muscle sympathetic responses to static handgrip and metaboreflex activation in young healthy men

**DOI:** 10.14814/phy2.13342

**Published:** 2017-07-18

**Authors:** Anthony V. Incognito, Connor J. Doherty, Jordan B. Lee, Matthew J. Burns, Philip J. Millar

**Affiliations:** ^1^ Department of Human Health and Nutritional Sciences University of Guelph Guelph Ontario Canada; ^2^ Department of Kinesiology University of Guelph‐Humber Toronto Ontario Canada; ^3^ Toronto General Research Institute Toronto General Hospital Toronto Ontario Canada

**Keywords:** Blood pressure, ischemic preconditioning, isometric exercise, sympathetic nervous system

## Abstract

Ischemic preconditioning (IPC) has been hypothesized to elicit ergogenic effects by reducing feedback from metabolically sensitive group III/IV muscle afferents during exercise. If so, reflex efferent neural outflow should be attenuated. We investigated the effects of IPC on muscle sympathetic nerve activity (MSNA) during static handgrip (SHG) and used post‐exercise circulatory occlusion (PECO) to isolate for the muscle metaboreflex. Thirty‐seven healthy men (age: 24 ± 5 years [mean ± SD]) were randomized to receive sham (*n* = 16) or IPC (*n* = 21) interventions. Blood pressure, heart rate, and MSNA (microneurography; sham *n* = 11 and IPC *n* = 18) were collected at rest and during 2 min of SHG (30% maximal voluntary contraction) and 3 min of PECO before (PRE) and after (POST) sham or IPC treatment (3 × 5 min 20 mmHg or 200 mmHg unilateral upper arm cuff inflation). Resting mean arterial pressure was higher following sham (79 ± 7 vs. 83 ± 6 mmHg, *P* < 0.01) but not IPC (81 ± 6 vs. 82 ± 6 mmHg, *P* > 0.05), while resting MSNA burst frequency was unchanged (*P* > 0.05) with sham (18 ± 7 vs. 19 ± 9 bursts/min) or IPC (17 ± 7 vs. 19 ± 7 bursts/min). Mean arterial pressure, heart rate, stroke volume, cardiac output, and total vascular conductance responses during SHG and PECO were comparable PRE and POST following sham and IPC (All *P* > 0.05). Similarly, MSNA burst frequency, burst incidence, and total MSNA responses during SHG and PECO were comparable PRE and POST with sham and IPC (All *P* > 0.05). These findings demonstrate that IPC does not reduce hemodynamic responses or central sympathetic outflow directed toward the skeletal muscle during activation of the muscle metaboreflex using static exercise or subsequent PECO.

## Introduction

Exposing tissue to repeated periods of brief circulatory occlusion, termed ischemic preconditioning (IPC), has been shown to protect the myocardium (Murry et al. 1986, 1990) and skeletal muscle (Pang et al. 1995; Addison et al. 2003) against subsequent ischemia–reperfusion injury. A growing number of studies have also reported that IPC may be used as an ergogenic aid to improve human exercise performance (for review: Incognito et al. 2016; Salvador et al. 2016). One mechanism hypothesized to account for the observed exercise benefits is reduced stimulation of metabolically sensitive small diameter skeletal muscle afferents (Crisafulli et al. 2011; Cruz et al. 2016, 2017; Salvador et al. 2016). Feedback from thinly myelinated group III/IV muscle afferents can induce central fatigue (Sidhu et al. 2014) and inhibit *α*‐motor neuron activation (Gandevia 2001; Amann et al. 2015) explaining prior observations of IPC‐mediated reductions in ratings of perceived exertion (Bailey et al. 2012; Cruz et al. 2015) and increases in electromyographic activity (Cruz et al. 2015, 2016) during exercise.

Group III/IV afferents also relay critical feedback regarding muscle stretch/tension (mechanoreflex) and metabolite accumulation (metaboreflex) to the brainstem to modulate the neurocardiovascular response to exercise, commonly referred to as the exercise pressor reflex (Mitchell et al. 1983; Victor et al. 1988; Ray et al. 1994). When stimulated, this peripheral reflex increases cardiac output and blood pressure through parasympathetic withdrawal and sympathetic activation (Mitchell et al. 1983; Victor et al. 1988; Ray et al. 1994). If IPC exerts its effects during exercise through a reduction in group III/IV muscle afferent activation, parallel changes in neural efferent responses should be evident. Indeed, forearm ischemia applied prior to intense rhythmic handgrip was shown to attenuate the rise in muscle interstitial norepinephrine (Costa et al. 2001); however, the effects on central sympathetic outflow are uncertain as this method does not measure the specific rate of norepinephrine release nor its clearance. The first study to use microneurography to quantify central sympathetic outflow directed toward the skeletal muscle reported that IPC blunted increases in muscle sympathetic nerve activity (MSNA) during early (4–8 min) and late (16–20 min) forearm ischemia but not during the reperfusion period (Lambert et al. 2016). Unfortunately, while prolonged forearm ischemia can increase metabolite accumulation (Harris et al. 1975), hemodynamic responses are more closely associated with perceptual measures of pain (Maixner et al. 1990). In contrast, IPC was reported recently to increase systemic vascular resistance during post‐exercise circulatory occlusion (PECO) (Mulliri et al. 2016), a technique commonly used to isolate the influence of the muscle metaboreflex (Mitchell et al. 1983; Victor et al. 1988; Ray et al. 1994). Accordingly, the effects of IPC on sympathetic outflow during exercise and isolated muscle metaboreflex activation are yet to be investigated.

Therefore, the purpose of this study was to investigate the effects of IPC on sympathetic outflow to skeletal muscle (MSNA) during static handgrip (SHG) exercise, a potent stimulator of the muscle metaboreflex (Mitchell et al. 1983; Victor et al. 1988; Ray et al. 1994), and isolated metaboreflex activation using PECO. We hypothesized that IPC would attenuate the MSNA burst frequency response (primary variable) during SHG exercise and PECO coincident with a reduction in mean arterial pressure.

## Methods

### Participants

Thirty‐seven healthy young (24 ± 5 years [mean ± SD]), nonobese (BMI: 24 ± 3 kg/m^2^) men were recruited to participate in the study. Recruitment was confined to men as women have been reported to have blunted ergogenic responses to IPC (Paradis‐Deschênes et al. 2016) and smaller exercise pressor responses to SHG and PECO (Ettinger et al. 1996). All participants were nonsmoking, in sinus rhythm, had no history of cardiovascular disease, and not taking any prescribed or over‐the‐counter medications. Participants were asked to abstain from vigorous physical activity for 48 h and from caffeine and alcohol for 24 h prior to the study visit to prevent blunting of the IPC response (Incognito et al. 2016). The University of Guelph Research Ethics Board approved all procedures and informed written consent was obtained from all participants prior to participation.

### Measurements

Heart rate was measured continuously using single‐lead electrocardiography (Lead II). A piezoelectric respiration belt (Model 1132 Pneumotrace II; UFI, Morro Bay, CA) was placed around the mid‐to‐upper abdomen to ensure participants maintained spontaneous breathing and avoided the Valsalva maneuver during exercise. Blood pressure was measured discretely at a minute‐to‐minute frequency from the left upper arm using an automated oscillometric sphygmomanometer (Model BPM‐200; BpTRU, Coquitlam, BC) and continuously (beat‐to‐beat) from the right third digit using an automated photoelectric plethysmography device (Finometer MIDI; Finapres Medical Systems, Enschede, The Netherlands). The continuous blood pressure and pulse wave recording permitted calculation of stroke volume using the Model Flow method (Wesseling et al. 1993), allowing determination of cardiac output and total vascular conductance.

Postganglionic efferent MSNA was measured continuously from the right fibular nerve using microneurography, as described previously (Notay et al. 2016). A recording microelectrode (2 M*Ω*; Frederick Haer, Brunswick, ME) was inserted percutaneously into a motor fascicle with a reference microelectrode placed 1–2 cm away. The raw nerve signal was amplified (75,000–99,000 times) and band‐pass filtered (700–2000 Hz) followed by signal rectification and integration with a 1 msec time constant (Nerve Traffic Analyzer, Model 662C‐4; Absolute Design and Manufacturing Services, Salon, IA). Confirmation of the MSNA signal was made by demonstrating increased activity in response to an end‐expiratory apnea and an absence of responsiveness to unexpected clapping or light stroking of the skin.

Continuous heart rate, breathing excursions, blood pressure, and multiunit MSNA data were acquired at a frequency of 1 kHz and the raw MSNA neurogram at 10 kHz using LabChart (Version 8; PowerLab; ADInstruments, NSW, Australia).

### Experimental protocol

All participants completed an introductory visit to be familiarized with the study procedures, after which they were randomized using an online random sequence generator (random.org) to either the sham (*n* = 16) or IPC (*n* = 21) treatment group. Participants were studied on a single visit in a light and temperature controlled laboratory following voiding. Upon entering the laboratory, anthropometric measurements were collected and participants rested supine on a comfortable bed. Two handgrip maximal voluntary contractions (MVC) were completed in their left hand (Model 78010, Hand Dynamometer; Lafayette Instrument, Lafayette, LA) separated by 30–60 sec of rest. The highest recorded force was designated as MVC.

Following instrumentation and a 10 min acclimatization period, continuous and discrete data were collected over a 7 min baseline period. Next, participants completed the first SHG and muscle metaboreflex test (PRE). This test consisted of collecting heart rate, blood pressure, and MSNA during 3 min of baseline, 2 min of 30% MVC SHG using the left hand, and 3 min of PECO. Sequestering local metabolites produced from exercise using PECO allows for isolation of the muscle metaboreflex without the confounding influences of the muscle mechanoreflex and feedforward central command (Mitchell et al. 1983; Victor et al. 1988; Ray et al. 1994). PECO was induced by inflating a manual sphygmomanometer to 220 mmHg in the left upper arm. After an 8 min rest period to allow hemodynamic and MSNA values to return to baseline, participants were administered sham or IPC in the left upper arm using a manual sphygmomanometer connected to a 11 cm wide pneumatic compression cuff (DS400 Aneroid Sphygmomanometer; D.E. Hokanson Inc, Bellevue, WA). Both protocols consisted of 5 min of cuff inflation at either 200 mmHg (IPC) or 20 mmHg (sham) followed by 5 min of cuff deflation. This cycle was repeated three times totaling 30 min. Participants in both groups were informed that both interventions may improve exercise tolerance. A second SHG and muscle metaboreflex test (POST) commenced 3 min after the IPC or sham treatment and was completed using the same protocol described above.

### Data analysis

Offline analysis of the integrated MSNA neurogram was performed using a custom LabVIEW program (Millar et al. 2015; Notay et al. 2016). Acceptance of a multiunit sympathetic burst was made based on three established criteria: (1) sharp positive triangular‐shaped deflection with a width shorter than one cardiac cycle; (2) a minimum signal to noise ratio of 3:1; and (3) alignment with the time‐shifted cardiac cycle (White et al. 2015). From the integrated multiunit MSNA signal, burst frequency (bursts/min), burst incidence (bursts/100 heartbeats), and total MSNA (burst frequency × mean burst area) were calculated. As MSNA burst area is impacted by the distance between the recording electrode and discharging fibers (White et al. 2015), limiting interindividual comparisons, total MSNA during baseline periods was set at 100% and the change during SHG and PECO periods expressed as a percent.

Resting hemodynamic and MSNA data were averaged over the 3 min baseline periods preceding PRE and POST SHG and metaboreflex tests. Baseline values were used to calculate the change in continuous variables during the first (SHG1) and second (SHG2) minute of SHG and the third (i.e., last) minute of PECO.

### Statistical analysis

Baseline between‐group participant characteristics were compared using unpaired *t*‐tests. Resting hemodynamic and MSNA variables were compared before and after sham and IPC conditions using a two‐way repeated measures ANOVA. Hemodynamic and MSNA responses during the metaboreflex tests were analyzed using a two‐within (condition [PRE vs. POST] and time [baseline, SHG1, SHG2, and PECO]) and one‐between (group [Sham vs. IPC]) repeated measures ANOVA. To probe the isolated effects of muscle metaboreflex activation, hemodynamic and MSNA variables during PECO were also examined with a two‐way repeated measures ANOVA. Significant differences were investigated using Bonferroni post hoc procedures with correction for multiple comparisons. All data were analyzed using GraphPad Prism 6 (GraphPad Software Inc, La Jolla, CA) and IBM SPSS Statistics 23 (Armonk, NY). A *P *< 0.05 was considered statistically significant. Data presented as mean ± SD, unless otherwise specified.

## Results

**Figure 1:**
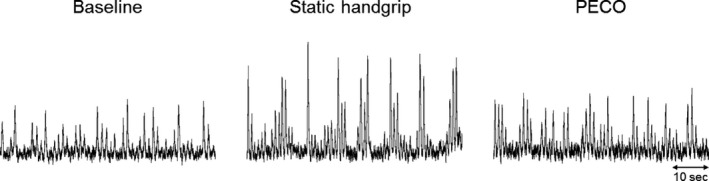
Representative multiunit muscle sympathetic nerve activity (MSNA) tracing from one participant during the last minute of baseline, static handgrip, and post‐exercise circulatory occlusion (PECO).

Baseline participant characteristics were similar between the sham and IPC group (Table 1). High‐quality MSNA recordings were obtained in 29 of 37 participants (sham *n* = 11; IPC *n* = 18), while in the remaining eight participants we were unable to locate a recording site. In those with microneurographic recordings (*n* = 29), the MSNA signal was lost in three participants after PRE and re‐established prior to POST (sham *n* = 2; IPC *n* = 1). A representative microneurographic recording is shown in Figure 1. Sham and IPC had no effect on resting hemodynamic and MSNA variables (All *P* > 0.05; Table 2), with the exception of mean arterial pressure which was higher following sham treatment (*P* < 0.01).

**Table 1:** Participant characteristics

Variable	Sham	IPC
Number, *n*	16	21
Age, years	23 ± 3	24 ± 4
Height, cm	177 ± 6	180 ± 6
Weight, kg	79 ± 11	77 ± 10
Body mass index, kg/m^2^	25 ± 3	24 ± 2
Handgrip MVC, kg	43 ± 10	49 ± 10
Systolic blood pressure, mmHg	109 ± 8	111 ± 6
Diastolic blood pressure, mmHg	64 ± 7	66 ± 6

Mean ± SD. MVC, maximal voluntary contraction; IPC, ischemic preconditioning.

**Table 2:** Resting hemodynamic and MSNA measurements before (PRE) and after (POST) sham and IPC treatment

Variable	Sham		IPC	
	PRE	POST	PRE	POST
Mean arterial pressure, mmHg	79 ± 7	83 ± 6^a^	81 ± 6	82 ± 6
Heart rate, beats/min	64 ± 10	63 ± 9	58 ± 8	60 ± 9
Stroke volume, mL	97 ± 12	98 ± 11	102 ± 14	102 ± 15
Cardiac output, L/min	6.2 ± 1.3	6.2 ± 1.0	5.9 ± 1.0	5.9 ± 0.9
Total vascular conductance, mL/min/mmHg	79 ± 14	74 ± 10	73 ± 13	72 ± 12
MSNA burst frequency, burst/min	18 ± 7	19 ± 9	17 ± 7	19 ± 7
MSNA burst incidence, bursts/100 heartbeats	30 ± 10	31 ± 15	31 ± 14	33 ± 12

Data obtained from 37 participants, with the exception of MSNA (*n* = 29). Mean ± SD. MSNA, muscle sympathetic nerve activity; IPC, ischemic preconditioning.

a *P* < 0.01 versus PRE of same group.

**Figure 2:**
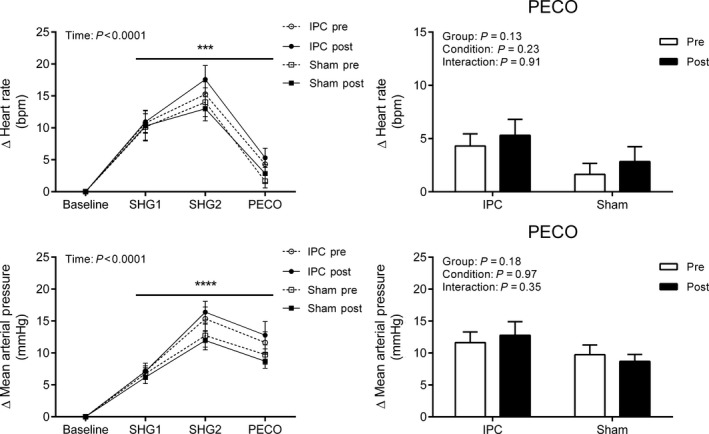
Change in heart rate and mean arterial pressure during the first and second minute of static handgrip (SHG1 and SHG2) and post‐exercise circulatory occlusion (PECO) before (PRE) and after (POST) sham (*n *=* *16) or IPC (*n* = 21). Values presented as mean ± SEM. Data assessed using a three‐way (left side) or two‐way (right side – PECO only) repeated measures ANOVA. ****P < 0.0001; ***P < 0.001 versus baseline.

**Figure 3:**
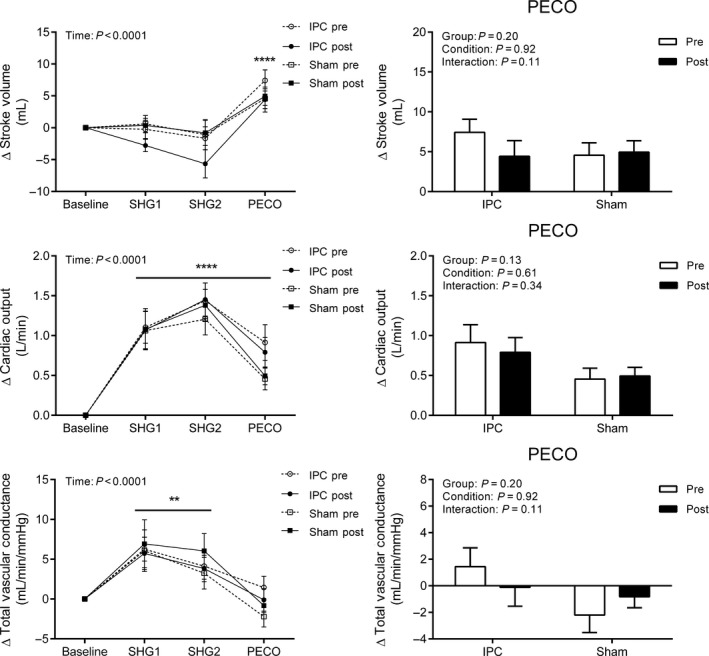
Change in stroke volume, cardiac output, and total vascular conductance during the first and second minute of static handgrip (SHG1 and SHG2) and post‐exercise circulatory occlusion (PECO) before (PRE) and after (POST) sham (*n* = 16) or IPC (*n* = 21). Values presented as mean ± SEM. Data assessed using a three‐way (left side) or two‐way (right side – PECO only) repeated measures ANOVA. ****P < 0.0001; **P < 0.01 versus baseline.

During SHG and PECO, all hemodynamic variables exhibited a main effect of time (All *P* < 0.001, Figs. 2 and 3). More specifically, the changes in heart rate, mean arterial pressure, and cardiac output were elevated from baseline during SHG1, SHG2, and PECO (All *P* < 0.0001), whereas total vascular conductance was increased only during SHG1 and SHG2, and stroke volume increased only during PECO (All *P* < 0.0001). There were no differences in the main effects for condition or group, or any of the interaction terms (All *P* > 0.05).

**Figure 4:**
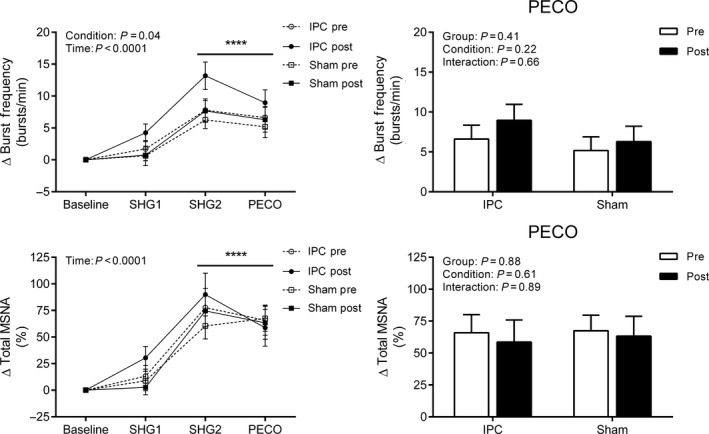
Change in muscle sympathetic nerve activity (MSNA) burst frequency and total MSNA during the first and second minute of static handgrip (SHG1 and SHG2) and post‐exercise circulatory occlusion (PECO) before (PRE) and after (POST) sham (*n* = 11) or IPC (*n* = 18). Values presented as mean ± SEM. Data assessed using a three‐way (left side) or two‐way (right side – PECO only) repeated measures ANOVA. ****P < 0.0001 versus baseline.

Similarly, there was a main effect of time across all MSNA variables (All *P* < 0.05, Fig. 4), where MSNA burst frequency, burst incidence (data not shown), and total MSNA were elevated from baseline during SHG2 and PECO. A main effect of condition was detected for MSNA burst frequency such that values were higher during POST (*P* < 0.05); however, no group or interaction effects were detected for any MSNA variables (All *P* > 0.05).

## Discussion

The present study is the first to investigate the effects of IPC on a direct measure of central sympathetic outflow during exercise and isolated muscle metaboreflex activation. In contrast to our hypothesis, IPC did not attenuate MSNA or pressor responses during submaximal SHG or PECO. Thus, these results do not support the capacity for IPC to attenuate the efferent neural response following activation of group III/IV skeletal muscle afferents.

It has been suggested that IPC may attenuate group III/IV muscle afferent feedback through the activation of opioid receptors (Cruz et al. 2016, 2017; Salvador et al. 2016). The cytoprotective effects of IPC against skeletal muscle ischemia–reperfusion injury are abolished by nonselective blockade of opioid receptors (Addison et al. 2003), while intrathecal administration of the *μ*‐opioid receptor agonist, fentanyl, attenuates spinal feedback from group III/IV skeletal muscle afferents (Hill and Kaufman 1990; Meintjes et al. 1995). However, it should be noted that exercise studies administering fentanyl have demonstrated time‐dependent effects on performance with early increases in central motor drive (benefit) and late increases in peripheral fatigue (detriment) (Amann et al. 2009; Blain et al. 2016; Hureau et al. 2017). Such divergent time‐dependent responses have not been reported previously following IPC. Instead, we hypothesized that IPC may reduce stimulation of metabolically sensitive skeletal muscle afferents based on evidence that IPC can alter skeletal muscle metabolism, reducing lactate accumulation in animals during prolonged ischemia (Murry et al. 1990; Pang et al. 1995; Addison et al. 2003) and in humans during submaximal exercise (Bailey et al. 2012), although the latter not consistently (Kido et al. 2015; Sabino‐Carvalho et al. 2017). Lower post‐exercise blood lactate levels following IPC have been shown recently to correlate with improvements in a self‐paced 5 km running time trial (Seeger et al. 2017). Lactic acid is a potent stimulator of metabolically sensitive group III/IV muscle afferents (Rotto and Kaufman 1988) and the inability to generate lactic acid abolishes the MSNA response to SHG and PECO (Fadel et al. 2003).

Unfortunately, only one study has examined the direct efferent neural response following IPC. Lambert et al. (2016) demonstrated that IPC attenuated increases in MSNA during prolonged forearm ischemia (but not reperfusion) in young healthy adults. Importantly, they noted blunted vasoconstrictor drive in the early phase (4–8 min) of ischemia, a period that is not associated with significant changes in metabolite concentrations (Harris et al. 1975). Prior work has linked hemodynamic responses with perceptual measures of pain (Maixner et al. 1990) suggesting that a reduction in pain perception may be responsible for the observed early and late attenuations in MSNA. Indeed, IPC has been shown to possess analgesic effects (Pereira et al. 2016), although not tested specifically in skeletal muscle. Notwithstanding that PECO procedures can also activate central pain pathways (Macefield and Henderson 2015), SHG at 30% MVC has been shown consistently to increase MSNA during the second minute of contraction coincident with increased metabolite accumulation (a more rapid response as compared to prolonged ischemia), which is maintained during isolation of the metaboreflex with PECO (Mitchell et al. 1983; Victor et al. 1988; Ray et al. 1994). Therefore, we feel that our experimental procedures are more sensitive to isolating the effects of IPC on the muscle metaboreflex compared to prolonged ischemia at rest.

In our study, IPC did not alter heart rate, mean arterial pressure, stroke volume, cardiac output, or total vascular conductance responses during SHG or PECO. Our findings during PECO are in contrast with a prior observation following rhythmic handgrip exercise (Mulliri et al. 2016), which demonstrated a blunted pressor response secondary to reductions in stroke volume and cardiac preload, as well as an increase in total vascular resistance. The reductions in stroke volume and mean arterial pressure may have unloaded the cardiopulmonary or arterial baroreflex, increasing peripheral vasoconstrictor outflow (MSNA) (Floras et al. 2001; Fu et al. 2006) and explaining the rise in total vascular resistance (Mulliri et al. 2016). The mechanisms responsible for a reduction in stroke volume or cardiac preload following IPC are not clear but have been suggested to involve nitric oxide‐mediated venodilation (Mulliri et al. 2016). IPC has been shown to increase nitric oxide bioavailability (Costa et al. 2001; Rassaf et al. 2014). The absence of a reduction in stroke volume in the present study may be influenced by the fact that participants were studied in the supine, as opposed to the seated (Mulliri et al. 2016) or upright (Fu et al. 2006) posture. Regardless, the lack of attenuated MSNA during SHG and PECO suggests that IPC does not alter feedback from group III/IV metabolically sensitive muscle afferents.

It is important to consider that two phenotypes of chemosensitive group III/IV skeletal muscle afferents exist (Pollak et al. 2014; Amann et al. 2015). Metaboreceptors, activated by low innocuous levels of metabolite accumulation, such as during freely perfused exercise, and metabonociceptors, activated by high noxious levels of metabolite accumulation, such as during ischemic exercise and PECO (Pollak et al. 2014; Amann et al. 2015). Although unclear in humans, data from mice suggest that metabonociceptors, but not metaboreceptors, possess acid‐sensing ion channel 3 (ASIC3) and transient receptor potential vanilloid 1 (TRPV1) receptors (Jankowski et al. 2013). The existence of nonoverlapping receptor populations offers the possibility, albeit speculative, that IPC may impact group III/IV afferent phenotypes differentially. This could explain how IPC alters central motor drive (Cruz et al. 2015, 2016) but not hemodynamic responses (present study). A mechanism for such a response is unclear and the phenotypic differences between metabonociceptors and metaboreceptors are not fully understood (Amann et al. 2015) but warrant further investigation.

We acknowledge several considerations. Our study population was restricted to young healthy men, limiting the generalizability of results to women, healthy aging, or clinical populations. As commonly used (Mitchell et al. 1983; Victor et al. 1988; Ray et al. 1994; Amann et al. 2015), PECO was considered to isolate the muscle metaboreflex; however, this methodological approach may preferentially isolate metabonociceptors (Amann et al. 2015). It must also be noted that the 20 mmHg sham procedure may represent a weaker control as compared to complete occlusion for 1 min (Ferreira et al. 2016) or therapeutic ultrasound (Sabino‐Carvalho et al. 2017), and therefore, placebo and nocebo effects may still have influenced results despite communication with the participants that both interventions may improve exercise tolerance. Further, the determination of an optimal IPC protocol for cytoprotection or exercise benefits in humans remains unknown. Thus, while we selected our IPC protocol based on its capacity to exert benefits on human exercise performance previously (Incognito et al. 2016; Salvador et al. 2016), our results may not extend to alternative protocols with a different number or duration of ischemia–reperfusion cycles. The short time period separating IPC administration and our exercise intervention may also impact the results (Lisbôa et al. 2017), although studies have demonstrated significant performance effects on cycling (de Groot et al. 2010; Crisafulli et al. 2011; Kido et al. 2015) and static leg extension (Tanaka et al. 2016) to failure utilizing similar short time periods. Additionally, no relationship was observed between the timing of IPC administration and exercise performance in a recent meta‐regression analysis (Salvador et al. 2016).

In conclusion, our results demonstrate that IPC does not attenuate central sympathetic outflow directed toward skeletal muscle or the pressor response following activation of metabolically sensitive group III/IV skeletal muscle afferents with moderate‐intensity SHG or subsequent PECO. Whether IPC has the capacity to differentially impact metabolically sensitive group III/IV muscle afferent subtypes or exert ergogenic effects through reductions in pain perception warrants further study.

## Conflict of Interest

None declared.
